# Evaluation of the Interaction Between Wharton’s Jelly-Derived Mesenchymal Stem Cells and β-Mercaptoethanol

**DOI:** 10.7759/cureus.83115

**Published:** 2025-04-28

**Authors:** Fatma Öz Bağcı, Aydan Özgörgülü, Gülsemin Çiçek, Emine Utlu Özen, Selçuk Duman, Tahsin Murad Aktan, Ismail Reisli

**Affiliations:** 1 Histology and Embryology, Faculty of Medicine, Necmettin Erbakan University, Konya, TUR; 2 Histology and Embryology, Etlik Zübeyde Hanım Women's Health Education and Research Hospital, Ankara, TUR; 3 Child Health and Diseases, Faculty of Medicine, Necmettin Erbakan University, Konya, TUR

**Keywords:** cell culture, flow cytometry, immunocytochemistry staining, stem cells, wharton jelly derived mesenchymal stem cell

## Abstract

Purpose: Wharton's jelly-derived mesenchymal stem cells (WJ-MSCs) are self-regenerative and able to differentiate into multipotent stem cells. There may be different sources of mesenchymal stem cells (MSCs) involved in the repair mechanism of damaged tissues in the organism. WJ-MSCs may differentiate into osteocytes, chondrocytes, adipocytes, and myocyte cells. Furthermore, MSCs show neuroprotective effects on neurons. Today, many MSC neuroregenerative treatments have been shown to be effective. Studies have shown that MSCs are more involved in paracrine effects due to their neuroprotective effect in multiple diseases such as multiple sclerosis, acute spinal cord injury and encephalomyelitis. The main aim of this study was to investigate the neuronal markers of stem cells after incubation with β-mercaptoethanol (BME).

Materials and methods: In our study, WJ-MSCs were thawed in a water bath at 37°C and cultivated in cell culture dishes. When the cell occupancy rate reached 60-70%, they were treated with 2 mM BME. At the first and third hours, MSCs were removed from the dishes, and flow cytometry and immunostaining revealed that BME's nestin, neuron filament light (NF-L), SOX1, SOX2, doublecortin (DCX), glial fibrillary acidic protein (GFAP), Ki67, and CD44 were evaluated.

Results: Immunocytochemically, nestin and NF-L values ​​of MSCs exposed to BME increased at the first hour. In the flow cytometric evaluation, it was observed that nestin was high in the first hour.

Conclusion: One of our aims in this study was to reduce the possible toxic side effects of BME for MSCs by exposing the BME used in previous studies at the minimum dose for neuronal differentiation. In our study, we showed first-hour changes similar to the neuronal differentiation obtained with pre- and post-induction in other studies.

## Introduction

For conditions such as graft-versus-host disease (GVHD), Crohn’s disease, kidney failure, diabetes, osteoarthritis, multiple sclerosis (MS), amyotrophic lateral sclerosis (ALS), intraventricular bleeding (IVK) in preterm infants, autism, cerebrovascular diseases, spinal cord injury, and trauma due to traffic accidents, there has been a search for new treatments and standardized approaches, which has brought the value of cellular treatments into focus [1,2].

The Wharton's jelly-derived mesenchymal stem cells (WJ-MSCs) used in our study are superior to other mesenchymal stem cells (MSCs) due to their ease of acquisition and high proliferation capacity [3]. These cells combine the characteristics of embryonic stem cells (ESCs) and adult stem cells. WJ-MSCs can express markers found in ESCs, such as Nanog, SOX2, and Oct-4, although to a lesser extent [4]. Studies have shown that WJ-MSCs are less exposed to external factors in the prenatal period than other stem cells, are obtained immediately after birth, and can be more effective in terms of differentiation and treatment [5]. MSCs can be obtained primarily from bone marrow and adipose tissue, as well as from umbilical cord blood, Wharton’s jelly, amniotic fluid, placenta, fetal tissues, synovium, endometrium, dental pulp, menstrual blood, salivary glands, and nasal cavities [6].

For stem cells to be applied in treatment, it is widely known that their stimulation must be facilitated before they reach their designated site to increase the number of cells that migrate to the damaged area and to define the microenvironment in which they will settle [7,8]. However, when the nervous system is damaged, cell regeneration becomes more challenging.

Stem cell therapies have potential clinical applications for neurodegenerative diseases. The use of differentiated stem cells from MSCs as an alternative to undifferentiated stem cells has been increasingly investigated over the past two decades to develop treatments for neurodegenerative diseases. Many studies have shown that MSCs can differentiate into neuronal cells and that they can be used in clinical treatments. Various stimulation protocols have been used for this purpose, especially for neurological stimulation and differentiation [9,10].

Neuronal differentiation is commonly induced using chemicals such as butyl hydroxyanisole (BHA), all-trans retinoic acid (ATRA), β-mercaptoethanol (BME), and valproic acid (VA). These compounds play a crucial role in directing stem cells toward a neurogenic lineage, enhancing their differentiation potential.

BME is a thiol-containing reducing agent known to modulate the intracellular redox state. It promotes early neuronal differentiation by altering the redox potential of cells, which in turn affects gene expression patterns related to neural lineage commitment. Specifically, BME increases intracellular glutathione levels, which can activate key signaling pathways involved in stem cell fate, including MAPK/ERK and PI3K/Akt pathways [11,12]. In addition, redox-sensitive transcription factors such as Nrf2 may also be involved in initiating neural differentiation cascades.

As a result of neurogenic differentiation, various stem cell markers are expressed. Nestin (NES) is highly expressed during cell division in the early stages of central nervous system (CNS) and peripheral nervous system development [13]. Another important marker, glial fibrillary acidic protein (GFAP), is primarily found in astroglia, which provide structural and metabolic support to neurons, particularly in response to neuronal damage [14].

In neuronal migration, doublecortin (DCX) plays a key role by stabilizing microtubules and facilitating polymerization, allowing immature neurons to move to their designated locations within the brain [15]. SOX1 is a major marker of neuroectoderm activation during gastrulation and is predominantly expressed in neuronal progenitor cells. Its expression declines during embryonic development, but it remains crucial for maintaining the progenitor pool [16,17]. Similarly, SOX2 is strongly expressed during implantation and epiblast stages and continues to be present in adult neurogenic regions, emphasizing its significance in neural development and regeneration [18,19].

Additionally, Ki67 serves as a proliferation marker, not only in MSCs but also in other proliferating cells, indicating active cell division and growth [20]. The expression of these markers provides critical insights into the differentiation process and potential applications in regenerative medicine and neurodegenerative disease research.

Our aim was to investigate the early neurogenic effects of BME on WJ-MSCs using a simplified and time-efficient protocol. Several studies have shown that BME can trigger early neuronal marker expression and morphological changes within 30 to 60 minutes of exposure. However, prolonged exposure durations and higher concentrations of BME have been associated with increased cytotoxicity and apoptosis. To this end, a minimal dose of 2 mM BME was applied for short durations (one and three hours) to minimize cytotoxicity while effectively inducing the expression of neuronal differentiation markers.

## Materials and methods

Ethical approval

This study was carried out with the approval of the Ethics Committee of the Necmettin Erbakan University Meram Faculty of Medicine (Decision No: 2019/1865).

Culture stage of stem cells

The cell line used in this study was human-derived umbilical cord connective tissue-derived MSCs (PCS-500-010, ATCC®, American Type Culture Collection, Manassas, USA). Specifically, P4 cells were used in the study. WJ-MSCs were centrifuged at 900 rpm for nine minutes to remove them from the dimethyl sulfoxide (DMSO) freezing medium by thawing and inoculated into 25 cm^2^ culture dishes (Nest, USA).

This stage and the subsequent culture processes were supplemented with 200 mM penicillin/streptomycin and L-glutamine (Mill Creek Life Sciences, USA) using Dulbecco's Modified Eagle Medium (DMEM) (Gibco-Life Technologies, USA) and 5% human platelet lysate (hPL; Capricorn, Germany) as the culture medium. Cultures were maintained in an incubator with a 5% CO_2_, 5% O_2_, and 90% N_2_ gas combination at 37°C. Two types of cultivation were performed for flow cytometry and immunocytochemical staining. For immunocytochemistry, the cells were inoculated into dishes with a cover slip at the bottom.

Treatment of stem cells with BME

After reaching 70-80% confluence in the culture dishes, the cells were divided into three groups. Study Group 1 was treated with BME for one hour, while Study Group 2 received BME treatment for three hours. In contrast, the control group was not exposed to BME, serving as a baseline for comparison.

The study groups were treated with 2 mM of BME (Pan Biotech, Germany) in the DMEM-penicillin mixture. At the end of the first and third hours, cells were detached using TrypLE™ Express (Gibco-Life Technologies, USA) for flow cytometric analysis and then lifted from the culture dish. Cells were transferred into conical-bottom tubes and centrifuged at 900 rpm for nine minutes. After centrifugation, the supernatant was discarded, and the remaining cell pellet was extracted for flow cytometric analysis.

At the first and third hours, cell viability was determined using trypan blue dye. The control group cells were also removed at the same time points without changing the medium, and flow cytometry and immunocytochemistry staining were performed.

Stem cell viability determination

The viability of the cells in all groups was evaluated using an automatic cell counter (Luna Automated Cell Counter, Logosbio, Korea) with trypan blue (Gibco-Life Technologies, USA) staining. A 1:1 ratio of 4% trypan blue was used to determine the cell viability rate.

Flow cytometry analysis

Flow cytometric analyses were performed for all groups at the end of the first and third hours to assess neuronal differentiation markers. Cells were prepared for flow cytometry using a neural lineage kit (BD, 561526, USA) according to the manufacturer’s staining protocols. The following markers were evaluated: Nestin, DCX, GFAP, SOX1, SOX2, CD44, and Ki67. The protocol was repeated five times, and the analyses were performed using an FACS CANTO II (BD Biosciences, USA) instrument.

Immunocytochemical staining

After all cells from each group were purified from the culture medium, they were fixed using 4% paraformaldehyde solution (Santa Cruz, USA) dissolved in phosphate-buffered saline (PBS) (Pan Biotech, Germany). The preparation was treated with hydrogen peroxide, followed by UV blocking (ThermoFisher, USA) using a staining kit.

Primary antibodies against NES, neurofilament light (NF-L), and GFAP (all from Abcam, UK; diluted 1:200) were applied and incubated at room temperature for one hour, followed by treatment with a secondary antibody (ThermoFisher, USA). After each step, the cells were thoroughly washed with PBS to remove any unbound antibodies. Staining of the markers was performed using the AEC substrate system (ThermoFisher, USA), and Mayer’s hematoxylin was applied to counterstain cell nuclei.

To finalize the preparation, cover slips were sealed with a mounting medium and allowed to dry. For quantitative analysis, an average of 200 cells was counted for each antigen in each group. Immunocytochemical staining was performed using NES, NF-L, and GFAP antibodies, and the stained samples were examined under an Olympus BX43 (Japan) light microscope for further analysis.

Statistical analysis

All statistical analyses were performed using one-way analysis of variance (ANOVA) to assess differences between experimental groups. Results are presented as mean ± standard deviation (SD), and a p-value of less than 0.05 was considered statistically significant. Statistical calculations and graphical representations were carried out using IBM SPSS Statistics for Windows, Version 29 (Released 2021; IBM Corp., Armonk, New York, United States).

## Results

Approximately 1.5 million cells were counted in 25 cm^2^ culture dishes across all groups. Dead cells were stained blue using trypan blue. The cell viability of Group 1, Group 2, and the control group was measured as 89.33 ± 1.53%, 88.00 ± 1.00%, and 94.00 ± 1.00%, respectively, as determined by the Luna automatic cell counter. These viable cells were subsequently utilized for flow cytometric analysis.

**Figure 1:**
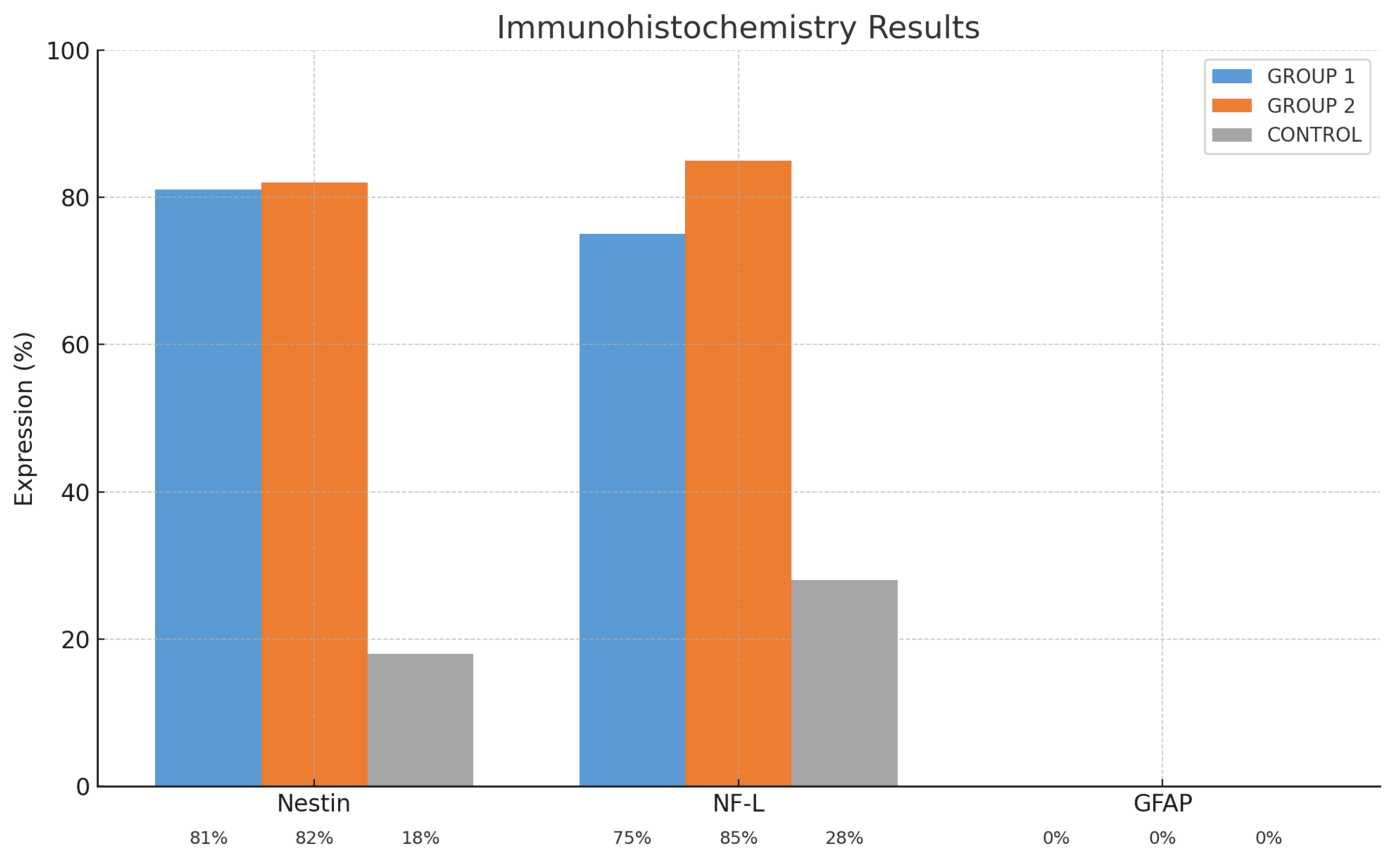
Immunohistochemistry (ICH) results NF-L: neurofilament light; GFAP: glial fibrillary acidic protein

**Figure 2:**
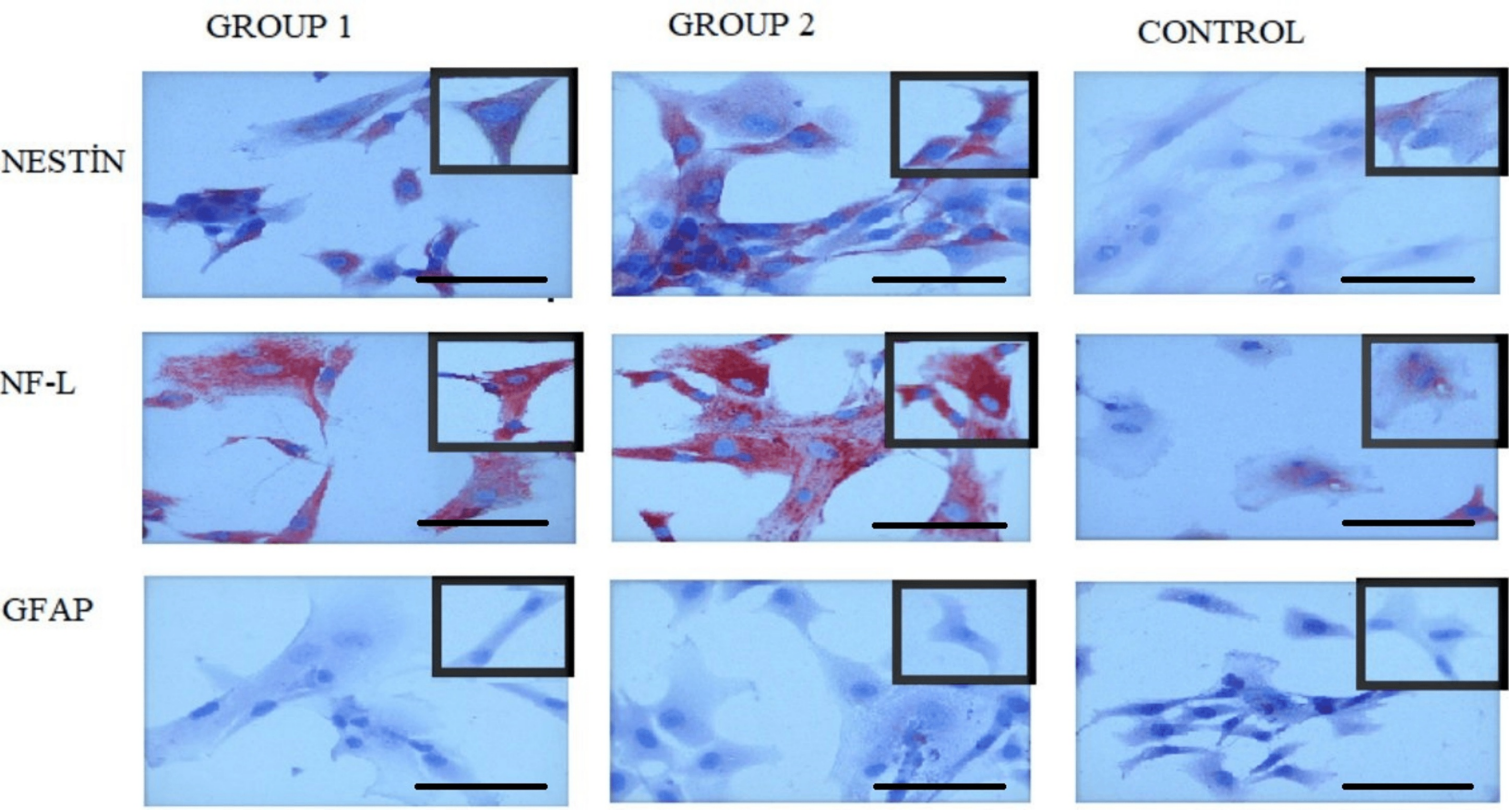
Immunocytochemistry (ICH) staining results of neurogenic markers in MSCs at 40x magnification (little images are enlarged versions of large images). MSCs: mesenchymal stem cells; NF-L: neurofilament light; GFAP: glial fibrillary acidic protein

At the end of immunocytochemical staining, an average of 200 cells were counted on each coverslip across all groups. AEC chromogen was used to detect cellular surface expression positivity, while Mayer’s hematoxylin was applied for nuclear staining (Figures 1-2).

**Figure 3:**
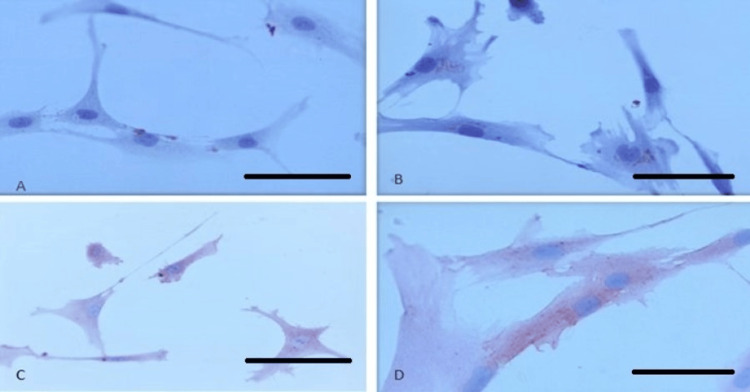
Immunostaining of CD surface markers in MSCs. (A) CD19-negative cells, (B) CD90-positive cells, (C) CD44-positive cells, and (D) CD105-positive cells. MSCs: mesenchymal stem cells

Simultaneously, MSC markers were assessed immunocytochemically in all groups. CD44 surface expression was observed as general cytoplasmic staining. CD90 positivity was detected as perinuclear staining. CD105 positivity was observed as cytoplasmic staining. According to MSC characterization criteria, MSCs should express CD105, CD44, and CD90, while lacking CD19 and other surface molecules (Figure 3).

Flow cytometry studies were repeated five times for each group, and the average values were calculated and presented as graphs. To evaluate the differences in marker expression levels among Group 1, Group 2, and the Control group, statistical analysis was performed. The mean ± standard deviation (SD) values for each group are presented in the accompanying table (Table 1). First, a one-way ANOVA was conducted to determine whether there were statistically significant differences among the groups. The ANOVA test revealed significant differences in the expression levels of multiple markers (p < 0.05). Subsequently, Tukey's Honestly Significant Difference (Tukey HSD) post hoc test was applied to identify specific group differences. All statistical analyses were performed with a significance threshold set at p < 0.05.

**Table 1:** Summary of mean ± standard deviation (SD) values for all experimental groups. DCX: doublecortin; GFAP: glial fibrillary acidic protein

Marker	Group 1 (Mean ± SD)	Group 2 (Mean ± SD)	Control (Mean ± SD)
Nestin	77.3 ± 0.71	77.4 ± 0.85	14.5 ± 0.71
DCX	91.85 ± 1.48	95.65 ± 2.62	52.3 ± 0.99
GFAP	0.45 ± 0.21	0.3 ± 0.42	0.0 ± 0.0
SOX1	97.65 ± 2.62	97.8 ± 2.83	96.25 ± 4.6
SOX2	98.15 ± 1.91	97.15 ± 0.49	46.0 ± 2.83
CD44	96.8 ± 4.17	97.25 ± 3.34	98.15 ± 1.06
Ki67	97.8 ± 0.99	98.8 ± 1.48	66.4 ± 3.32

**Figure 4:**
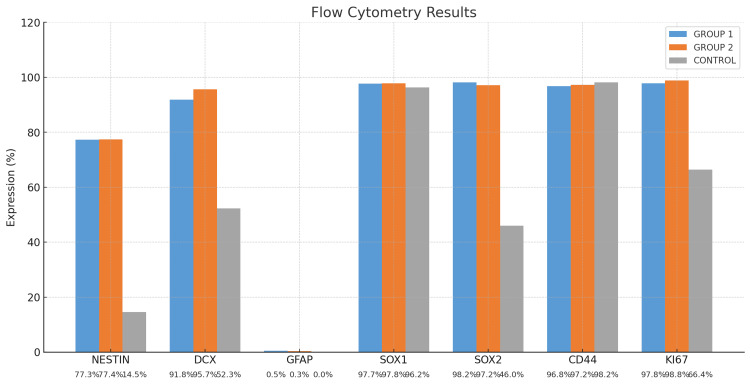
Flow cytometry results DCX: doublecortin; GFAP: glial fibrillary acidic protein

**Figure 5:**
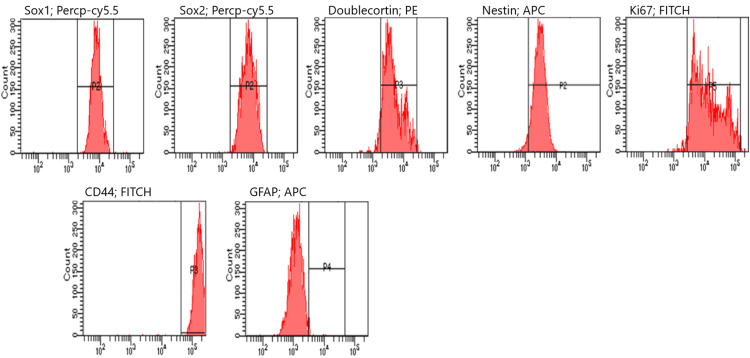
Flow cytometry analysis at the first hour. GFAP: glial fibrillary acidic protein

**Figure 6:**
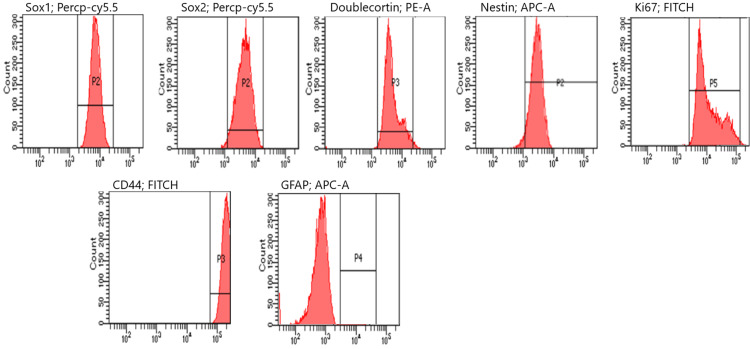
Flow cytometry analysis at the third hour. GFAP: glial fibrillary acidic protein

**Figure 7:**
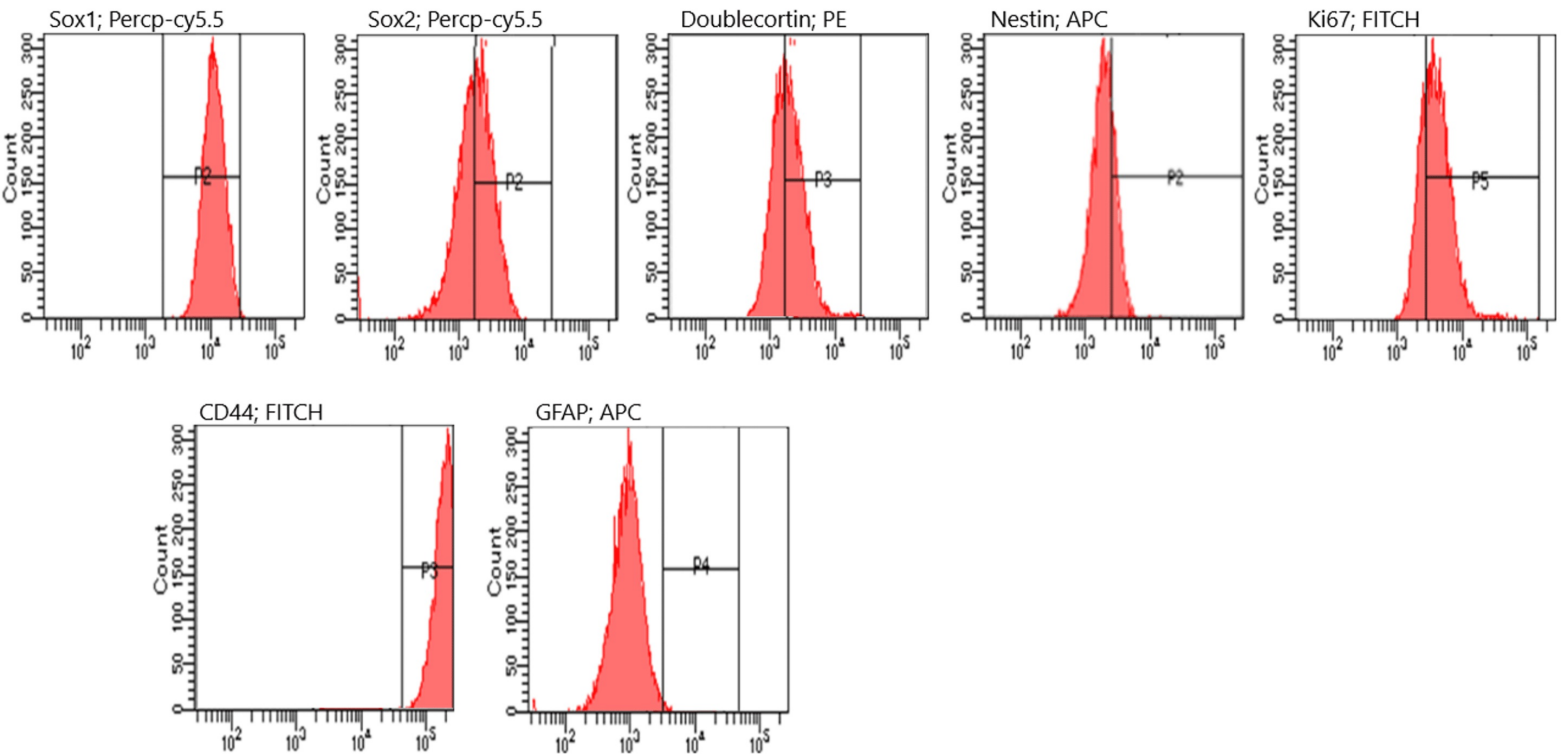
Flow cytometry analysis of the control group. GFAP: glial fibrillary acidic protein

Flow cytometry analysis revealed that, following a one-hour treatment period, NES expression significantly increased from 15% in the control group to approximately 77% in the treated group (p < 0.05). Similarly, DCX, SOX2, and Ki67 levels exhibited substantial upregulation, with mean increases of nearly 50% compared to the control group. These changes were statistically significant (p < 0.05), indicating enhanced neural differentiation and proliferative activity in the treated cells (Figure 4). The results of one analysis of each group from flow cytometry experiments are shown (Figures 5-7).

## Discussion

Although WJ-MSCs exhibit certain features of ESCs, many studies have been conducted to determine why they do not form teratomas and can be used as autologous-allogeneic cells. Some of these studies have focused on neurological disorders [21], kidney damage, lung injury [22], orthopedic injuries [23], and liver damage, demonstrating that WJ-MSCs can be utilized in multiple therapeutic areas. The absence of an autologous and allogeneic immune response in both preclinical and clinical studies was one of the key reasons why WJ-MSCs were selected for this study.

In addition to their well-known ability to differentiate into adipogenic, osteogenic, and chondrogenic lineages, the potential of MSCs to differentiate into neuronal cells has also been widely investigated. This differentiation has been achieved using pre-induction, induction, and post-induction protocols, either individually or in combination. In these studies, NES, NF-L, and MAP2 were evaluated as neuronal markers [24,25].

A study by Ahmadi et al. utilized a two-step chemical induction protocol involving both pre-induction (1 mM BME for 24 hours) and post-induction (up to 10 mM BME for one to five days) phases to induce neural differentiation in adipose-derived stem cells (ADSCs), reporting 87% NES positivity on the first day after induction. However, this approach also led to a marked decline in cell viability by day 3 (0.47 ± 0.01%, p < 0.001), indicating significant BME-induced cytotoxicity and apoptosis (Table 2) [26]. In contrast, our study employed a single one-hour BME exposure without any pre- or post-induction steps and achieved 77% NES positivity. Interestingly, no significant differences were observed between the first and third hours post-treatment, suggesting that early commitment may occur rapidly. Moreover, cells treated with BME in our protocol retained comparable confluency after repassage, similar to untreated controls, indicating a lower level of cytotoxic stress. These findings suggest that short-term BME exposure may be sufficient to induce early neural marker expression while preserving cell viability and warrant further investigation into long-term differentiation outcomes and later time points.

**Table 2:** Examples of studies to induce neuronal differentiation of MSCs. MSCs: mesenchymal stem cells; WJ-MSCs: Wharton's jelly-derived stem cells; DMEM: Dulbecco's Modified Eagle Medium; MEM: Minimum Essential Medium; BME: β-mercaptoethanol; DMSO: dimethyl sulfoxide; BHA: butyl hydroxyanisole; NF-L: neurofilament light; GFAP: glial fibrillary acidic protein

Author name	Origin of MSCs	Protocols	Normal culture medium	Pre-induction	Post-induction	Marker positivity immunohistochemistry (ICH)	RT-PCR	Additional findings
Ahmadi et al. [26]	Adipose-derived mesenchymal stem cells (AdMSCs)	Protocol 1	DMEM + 10% Fetal Bovine Serum (FBS)	DMEM + 20% FBS + 1 mM BME (24 hour)	DMEM + 2% DMSO + 1-10 mM BME + 200 µM BHA (1-5 days)	On day 1: 87% nestin+, 91% MAP2+, 70% GFAP+	Nestin, GFAP and MAP2 were shown on day 1, but it was thought not to be shown on day 3 due to apoptosis.	Apoptosis detected.
	-	Protocol 2	DMEM + 10% FBS	DMEM + 2% B27 + 20 ng/ml bFGF + 20 ng/ml hEGF (6-7 days)	5% FBS + 1% L-Glutamine + 1% non-essential amino acid + 1% N2 + 2% B27 (1 week)	On day 3: 51% nestin+, 80% MAP2+, 71% GFAP+	Markers were checked at 1 and 3 weeks. Nestin was higher at Week 1, while MAP2 was significantly higher at Week 3 GFAP fixed.	-
Abdullah et al. [27]	Bone marrow- mesenchymal stem cells (BM-MSCs)	-	MEM + 20% FBS + 1% ampicillin/streptomycin	MEM + 20% FBS + 1 mM BME (24 hour), medium cleaned; MEM + 2 mM BME (1 hour)	MEM + 1 mM RA + 10 ng/ml Neural Growth Factor (NGF) + 0.1 ng/ml sonic hedgehog (4 days)	MAP2 and choline acetyl transferase positivity were shown at the end of the study	-	Morphological changes were shown from the first 24-hour stage.
Drela et al. [20]	BM-MSC WJ-MSC	-	MSC Lonza medıum	They were exposed to 5% and 21% O2. Markers were checked at the end of each passage.	-	WJ-MSCs Ki67: 60%, while BM-MSC Ki67: 25% in hypoxic environment. WJ-MSC Nestin: 68% in early passages, while BM-MSC Nestin: 5% Nestin positivity decreased in later passages. WJ-MSC GFAP: 51%	It has been shown that the expression of factors supporting neurogenic progression such as VEGF, BDNF, GDNF, HGF, NT3 and NT4 is greatly increased in WGd-MSCs.	-
Mohammad et al. [11]	BM-MSC	-	MEM + 20% FBS + 100 mg/ml ampicillin/streptomycin	MEM + 20% FBS + 1 mM BME (24 hour)	MEM + 5 mM BME (10 hour)	NES at 24 hours: 61% NF-L: 32% NES at 27 hours: 86%, NF-L: 80% MAP: 29% positivity was shown at the 29th hour. Control group: NES: 10%, NF-L: 15%	In RT-PCR, NES positivity was shown 24 hours after exposure.	-
Woodbury et al. [12]	BM-MSC	-	αMEM + 20% FBS + 100 mg/ml ampicillin/streptomycin	DMEM + 20% FBS + 1 mM BME (24 hour)	DMEM + 1-10 mM BME/2% DMSO/200 µM BHA (6 days)	While it was nestin+ at the 30th minute after postinduction, it could not be detected on the 6th day. trkA was detected minimally at the 5th hour, and its expression was found to be increased at the 6th day.	-	-
Mohammad et al. [28]	BM-MSC	Protocol 1	MEM + 20% FBS + 500 µg/ml ampicillin/streptomycin	MEM + 20% FBS + 10 ng/ml bFGF (24 hour)	MEM + 2% DMSO + 200 µM BHA (5 days)	During post-induction: nestin: 61.2% (at 48 hour), NF-L: 86.7% (at 144 hour)	-	-
	-	Protocol 2	MEM + 20% FBS + 500 µg/ml ampicillin/streptomycin	MEM + 20% FBS + 1 mM BME (24 hour)	MEM + 5 mM BME (10 hour)	During post-induction: Nestin and NF-L highest at the 27th hour (86.3%, 80.8%)	-	-

A study by Mohammad et al. investigated bone marrow-mesenchymal stem cell (BM-MSC) differentiation and observed neuronal changes following BME treatment, with increasing effects at higher molarity. Immunocytochemical analysis revealed that NES expression increased from 10% to 60% at 24 hours, though subsequent increases were less pronounced. For NF-L, a significant increase was observed from 15% to 65% at the 25th hour, while MAP2 expression rose from 0% to 25% at the 29th hour.

Additionally, mRNA analysis in the same study indicated that NES and MAP2 peaked significantly at the 24th hour, followed by a decline. The highest NF-L expression was detected at the 27th hour (Table 2) [11]. In contrast, our study demonstrated the most significant increase in NES in the first hour, which was even higher than the levels reported by Mohammad et al.

Similarly, Woodbury et al. examined rat and human BM-MSCs using preinduction and induction models with BME and BHA. Immunocytochemical analysis showed NES marker positivity as early as 30 minutes after exposure to serum-free DMEM and BME. Neuronal morphology changes were observed within the first 60 minutes, with progressive alterations continuing over the course of three hours, indicating neuronal differentiation (Table 2) [12].

Unlike other studies, our research also examined SOX1, SOX2, Ki67, and CD44 expression. While SOX2 expression was low in the control group, it was significantly increased in the BME-treated groups, suggesting that the WJ-MSCs used in this study exhibited neural progenitor characteristics. Additionally, CD44 expression remained positive across all groups, indicating that BME treatment did not negatively impact MSC markers. While DCX expression was low in the control group, its increase in the BME-treated groups further supports neurogenic differentiation.

A study by Drela et al. demonstrated that hypoxia (5% O_2_) increased Ki67 expression from 40% to 60% in their neurotherapeutic evaluation of WJ-MSCs and BM-MSCs (Table 1) [20]. Similarly, in our study, Ki67 positivity was 68% in the control group but increased to 98% in Group 2, suggesting high proliferation rates.

Other studies have also highlighted the role of ATRA and UDP4 in the neuronal differentiation of MSCs [29]. According to Park et al., extracellular vesicles (EVs) derived from ADSCs exhibited neurogenic and neuroprotective effects, facilitating the differentiation of EVs into neuronal progenitor cells [30].

In this study, the first- and third-hour time points were deliberately selected to investigate the early cellular responses to BME stimulation. Our aim was to observe initial changes in neuronal marker expression while minimizing potential cytotoxic effects associated with prolonged exposure. Previous studies have shown that BME can induce neural morphological changes and marker expression within 30 to 60 minutes [12], making these early time points particularly relevant. Furthermore, cell viability analysis in our study showed that viability remained high - 89% at one hour and 88% at three hours - indicating that short-term exposure to 2 mM BME was well tolerated by the WJ-MSCs. These findings suggest that early exposure is sufficient to induce neurogenic commitment without compromising cell health.

Study limitations

This study has several limitations that should be acknowledged. First, the evaluation was limited to early time points (one and three hours) after BME treatment. This was a deliberate choice in order to focus on the immediate neurogenic responses and to minimize cytotoxic effects associated with prolonged exposure. As such, long-term differentiation or phenotypic stability was not assessed. Second, only a minimal dose of 2 mM BME was used, based on previous studies suggesting that lower concentrations reduce cellular stress while still initiating differentiation. Dose-response analysis was not performed and will be considered in future studies. Third, although key neuronal markers such as NES, NF-L, and GFAP were evaluated via immunocytochemistry and flow cytometry, gene expression analyses (e.g., reverse transcription-polymerase chain reaction (RT-PCR)) were not included, which may have provided additional molecular confirmation. Such analyses are planned to be incorporated in future studies to further support the protein-level findings.

The study exclusively used BME as the neuronal differentiation inducer. A comparison with other neurogenic inducers, such as retinoic acid (RA) or VA, could help determine the most effective differentiation strategy. The study focused solely on WJ-MSCs without comparison to other MSC sources (e.g., BM-MSCs or adipose-derived MSCs). A comparative analysis could provide a broader understanding of MSC neurogenic potential. In our future studies, we will plan new research to address and improve these limitations.

## Conclusions

One of our aims in this study was to reduce the possible toxic side effects of BME for MSCs by exposing the BME used in previous studies to the minimum dose for neuronal differentiation. In our study, we showed first-hour changes similar to the neuronal differentiation obtained with pre- and post-induction in other studies. This information shows that no consensus has yet been reached about which chemical to use, when to use it, or the confidence interval for differentiation in the neurogenic direction. The ability of MSCs to differentiate into neural lineages makes them a promising potential therapy to generate new advances in cell therapy and regenerative medicine. However, differentiation may vary depending on the method used, the site of collection, the physiological state, and the age of the individual from which they are isolated. There is a need for up-to-date research and procedures that can assist in the development and design of new therapies for the treatment of neurological and neurodegenerative diseases.

In summary, this study demonstrated that short-term exposure (one and three hours) to 2 mM BME is sufficient to induce early neuronal differentiation in WJ-MSCs while maintaining high cell viability. A significant increase in NES expression, from 15% to 77%, was observed within the first hour, indicating rapid neurogenic commitment. Other key neuronal and progenitor markers, including NF-L, SOX2, DCX, and Ki67, were also upregulated in the early phases following BME treatment. Importantly, cell viability remained above 88% in all treated groups, suggesting that this low-dose, short-duration protocol is both effective and less cytotoxic compared to previously reported induction methods. These findings support the potential use of BME as a rapid and safe inducer for neural differentiation in MSC-based therapies.
